# Gleanings from the German

**Published:** 1875-05-15

**Authors:** H. Gradle


### GLEANINGS FROM THE GERMAN.

BY DR. H. GRADLE.

### ON INTRODUCING NEEDLE ELECTRODES INTO THE HEART OF A DOG.

VULPIAN (*Arch. de Phys.*, 1874, p. 975–80) found the induced current to produce muscular tremors in both ventricles, changing, after four to five minutes, into fibrillary contractions until, after the further lapse of three to four minutes, a permanent diastolic arrest occurred. The result was the same, if the current had a duration of but one to two seconds, and it was not prevented by the use of chloral or curare. After the occurrence of the diastolic arrest, the cardiac muscle did not react to any excitation, direct or reflex. Neither section of the vagus, nor its paralysis by atropine, altered this result, which occurred also if only one electrode was inserted into the heart, the other being placed on some point of the cutaneous surface. Only on the use of weak currents did the irritability of the heart return. From this Vulpian concludes that the electric stimulus produces a spasm of the ventricles, preventing them from responding rhymthically to the impulses from the cardiac ganglia. This spasmodic contraction soon exhausts the irritability of the muscle. Vulpian therefore cautions the reader as to the feasibility of employing this method as means of resuscitation in man in cases of sudden syncope, for instance, from the use of chloroform.—*Centralbl. f. Chir.*, No. 15, 1875.

Desormeaux resorts to nitrate of silver after evacuation of the fluid in hydrocele, by introducing a stick of that substance through the canula of the trocar, and cauterizing the tunica vaginalis at several places. He claims a prompt inflammatory reaction, comparatively little pain, and perfect safety of the procedure. Also Maisoneuve and others are said to employ this method.—*Revue de Therap. Med Chir.*, 1875, No. 4.—*Centralbl. f. Chir.*, No. 15, 1875.

### ALTERATIONS OF SENSIBILITY IN ARTICULAR RHEUMATISM AND THE ELECTRO-THERAPEUTICS OF THIS DISEASE.

(*Centralbl. f. d. Med. Wissensch.*, No. 17, 1874).

For some time Drosdorf has had occasion to observe at Prof. Botkin's clinic (St. Petersburg) the following peculiar phenomenon in acute articular rheumatism: The sensibility to the electric current is reduced very decidedly in this disease, although the pain produced by moving or pressing upon the inflamed joints is quite considerable. This fact appears to overthrow the hypothesis of physiologists that all impressions are perceived by one apparatus. This observation induced the author to study the changes in the tactile and thermic senses as well as the sense of pressure and pain in affected joints, noting at the same time the altered electro-cutaneous sensibility, and the influence of faradization on the temperature of the joints.

For the latter purpose the author employed the apparatus of Du Bois Reymond, with 10,229 turns on the secondary coil with a Grove's element. One electrode consisted of a round copper disc of one and one-half inches diameter, the other a copper cylinder of somewhat smaller diameter, with sponge attached. The electrodes were moistened with a solution of common salt, and pressed against the skin. Thereupon the distance between the primary and secondary spiral was noted, giving the minimum (very weak current) and the maximum (pain). The faradization was continued for five minutes.

The tactile sense was determined by means of a pair of compasses after Weber's method, while the sense of pressure was studied by means of weights. In order to investigate the sense of temperature, the author placed upon the joint two metal discs previously dipped into water of different temperatures, ascertained with a centigrade thermometer. The temperature of the joints themselves was measured with a Geissler's thermometer, the reservoir of which consists of a narrow tube, bent on the horizontal plane and covered with a glass capsule, thus coming in contact with a larger surface than the ordinary instruments. The thermometer is slightly pressed against the skin, and in ten to fifteen minutes the mercury has attained its maximum height. In this manner Drosdorf examined several cases, and came to the following conclusions:

1. The sensation of pain consequent to the electric irritation is much reduced in the affected joints, sometimes even entirely gone, so that no pain is perceived when the coils are closely approximated, and by closure of the current numerous sparks are produced. At the same time pressure causes intense pain. The reduction of electro-cutaneous sensibility appears mostly proportional to the intensity of the disease and the pain produced by contact.

2. The diminution of electro-cutaneous sensibility is strictly limited; whenever a joint is affected only in part, the corresponding surface only loses its sensibility to the current.

3. This limit is not gradual, but abrupt.

4. This diminution of electro-cutaneous sensibility sometimes precedes the pain by two or three days, occasionally remaining after the latter has passed away.

5. If the electro-cutaneous sensibility has not yet become normal, the disease, though apparently cured, threatens to return.

6. Synchronously with this alteration, the sense of pressure is reduced, so that a weight of twenty to thirty grammes (five to seven and a half drachms) may not be perceived.

7. The sense of temperature is rendered more acute; the patient can recognize easily a difference of but 0.2° to 0.5° C. (0.36° to 0.90° F.)

8. The tactile sense of the skin covering the joints is heightened. In one case the patient could perceive over the diseased vertebral joints a distance of 0.2 to 0.3 centimetres between the points of Weber's pair of compasses. Sometimes a perversion is noted, one point producing the impression of two.

9. The heightened tactile and thermic sense is reduced by five to ten minutes' faradization.

10. The cutaneous temperature is always higher by 2–3° C. than over the corresponding joints of the normal side.

11. This increase of temperature often precedes and sometimes outlasts the rheumatic pains.

12. After five to ten minutes' faradization, the temperature becomes normal or even lower.

13. The rheumatic pains, increased by pressure and motion are diminished by faradization, so that a most sensitive joint may then permit of active or passive exercise.

14. The abatement of both the rheumatic pains and the temperature persists for three, four, or even five hours after faradization, thereupon gradually returning to the former point. But the duration of the paroxysms, as well as their intensity, is reduced.

15. Although the rheumatic process may run a shorter course, or annoy the patient less under the influence of faradization, still relapses occurred in one case, but the attacks became shorter in duration and less intense.

From all this it can be concluded that faradization for five to ten minutes daily will reduce the intensity of articular rheumatism, bring back the sensibility to the normal point, and lower the temperature of the affected joints. Some of the patients received no other treatment, and still recovered rapidly.

ARSENIC IN HEART DISEASE.

*Le Mouvement Medicale*, of March 13th, 1875, contains an article by Dr. L. Bonyer, according to which the therapeutic application of arsenic would be vastly increased, as he claims to cure, by means of the same, organic lesions of the heart, which so far we have only been able to palliate symptomatically with digitalis, almost our only medicament in this affection. The latter, generally efficacious in the primary functional disturbances of the circulation, is, according to universal experience, inert, if not harmful, in the latter stage, as it favors the stasis of blood, and hence the serous exudations. In fact, the action of digitalis is purely functional.

Arsenic, however, as Dr. Bonyer claims, does not only regulate the cardiac action, but also influences the anatomical changes; its effects on muscular and fibrous tissues are sufficiently demonstrated in rheumatism and gout. He administers the remedy in the form of "arsenical milk," a composition obtained by the action of arsenic (arsenious?) acid on the salts contained in whey. His usual dose is half a teaspoonful of the powdered arsenical milk, corresponding to about one centigramme (1–7 gr.) of arseniates. This he gives morning and evening, with eight to ten drops of tr. digitalis. From a number of cases, showing the palliative, if not curative effects of the agent, we select the following:

In the case of a man, æt. 50 years, affected with lesion of the aortic valves, the murmurs have disappeared after one month's treatment. In another remarkable case of a man of 45 years, with obstruction of the aortic valves, the existing cyanosis, dyspnœa, ascites, palpitation, and hematysis, have entirely disappeared after three months' treatment, an augmented impulse being now the only symptom. —*Allg. Wiener Med. Zeit.*, No. 13, 1875.

